# Obstructive sleep apnea risk is associated with a diminished cognitive profile: findings from the Canadian longitudinal study on aging

**DOI:** 10.3389/fpsyg.2026.1864558

**Published:** 2026-08-31

**Authors:** Dorrie Rizzo, Marc Baltzan, Mohamed Abu-Farha, Abdulmohsen Alterki, Jehad Abubaker, Fahd Al-Mulla, Sherif M. Elsaraj

**Affiliations:** 1 McGill University, Montreal, QC, Canada; 2 Lady Davis Institute for Medical Research, Montreal, QC, Canada; 3 Faculty of Medicine, McGill University, St Mary’s Hospital, Montreal, QC, Canada; 4 Department of Biochemistry and Molecular Biology, Dasman Diabetes Institute, Dasma, Kuwait; 5 Consultant Otolaryngology, Head & Neck Surgery, Department Otolaryngology, Head & Neck Surgery, Zain & Al Sabah Hospitals, Dasma, Kuwait; 6 Translational Research Department, Dasman Diabetes Institute, Dasma, Kuwait; 7 Faculty of Dental Medicine and Oral Health Sciences, McGill University, Montreal, QC, Canada

**Keywords:** cognitive impairment, insomnia, memory, obstructive sleep apnea, reaction time methods

## Abstract

**Background:**

Obstructive sleep apnea (OSA) and insomnia are highly prevalent in older adults and both have been linked to cognitive decline. However, it remains unclear whether the physiological burden of OSA or the sleep fragmentation associated with insomnia more strongly predicts cognitive impairment. This study examined whether OSA risk or insomnia better was associated with memory problems and cognitive slowing among community-dwelling older Canadians. Given the cross-sectional design, the study evaluated associations rather than causal relationships.

**Methods:**

Data were drawn from 946 participants in the Canadian Longitudinal Study on Aging (CLSA), including 473 individuals reporting a physician-diagnosed memory problem and 473 age- and sex-matched controls. OSA risk was assessed using the validated STOP-BA(N)G questionnaire. At the same time, insomnia was defined using operational research criteria based on sleep onset latency, wake after sleep onset, and duration. Cognitive processing speed was measured via Choice Reaction Time (CRT). General linear models, logistic regression, and discriminant analyses examined associations between sleep parameters and cognitive outcomes, controlling for age, sex, body mass index, income, and diabetes.

**Results:**

Participants with memory problems had significantly higher STOP-BA(N)G scores (*F*(1,931) = 7.10, *p* = 0.008) and slower CRTs (*p* = 0.05) compared to controls. Insomnia alone or comorbid with OSA (COMISA) was not associated with cognitive outcomes. In discriminant analysis, STOP-BA(N)G score (*r* = 0.45) and diabetes (*r* = 0.26) were the strongest predictors of memory problems, correctly classifying 66.6% of cases. Sex-stratified analyses demonstrated that the association between higher STOP-BA(N)G scores and memory problems remained significant in both men and women, although effect sizes were somewhat larger in men.

**Conclusion:**

Higher OSA risk was associated with poorer memory and slower cognitive processing in older adults, whereas insomnia was not associated with these outcomes. Because of the cross-sectional design, causality cannot be inferred. Screening for OSA and metabolic comorbidities in patients with memory complaints may help identify individuals at increased risk of cognitive impairment.

## 1 Introduction

OSA and insomnia are among the most common sleep disorders in the general population, particularly in older adults, who represent the fastest growing demographic group in Canada. The prevalence of undiagnosed OSA in this population may reach up to 80%, while chronic insomnia affects approximately 40% of older individuals (Morin et al., 2011). OSA is characterized by recurrent upper airway collapse leading to intermittent hypoxia, arousals, and fragmentation of sleep architecture (Punjabi, 2008; Eckert et al., 2009; Eckert and Butler, 2016). These pathophysiological changes contribute to oxidative stress, sympathetic activation, and systemic inflammation mechanisms implicated in cardiometabolic dysfunction and cognitive decline (Carreras et al., 2014; Dewan et al., 2015; Chasens et al., 2007; Cowie et al., 2015; Marshall et al., 2008; Alterki et al., 2023).

Emerging neuroimaging and neurophysiological evidence indicate that OSA can lead to structural and functional brain alterations, particularly in regions supporting memory and executive function, and that timely treatment may reverse some of these changes (Canessa et al., 2011; Owen et al., 2018). Insomnia, by contrast, is primarily associated with hyperarousal and sustained sleep fragmentation in the absence of hypoxia. Although it affects nearly 9–10% of Canadians and often co-occurs with OSA (COMISA), its independent contribution to cognitive impairment remains less clear (Rizzo et al., n.d; Sweetman et al., 2021). Patients with COMISA have been reported to experience greater distress and poorer sleep quality, yet the extent to which this comorbidity amplifies cognitive risk is uncertain.

Both OSA and insomnia have been associated with deficits in memory, attention, and executive function, as well as with increased vulnerability to neurodegenerative processes (Beaudin et al., 2021; Weihs et al., 2021). OSA has been associated with cognitive impairment, including mild cognitive impairment (MCI) and dementia, through mechanisms proposed in the literature such as intermittent hypoxia and sleep disruption. Approximately 27% of individuals with MCI also have OSA, highlighting the substantial overlap between sleep-disordered breathing and cognitive dysfunction (Beaudin et al., 2021; Weihs et al., 2021).

Despite growing evidence, the specific and comparative roles of OSA and insomnia in predicting cognitive outcomes remain underexplored in large, community-based samples. Many prior studies have relied on clinical cohorts, small samples, or single-center data, limiting generalizability. The Canadian Longitudinal Study on Aging (CLSA) provides an exceptional opportunity to address this gap by leveraging a large, nationally representative cohort with standardized measures of sleep, cognition, and health (Raina et al., 2009; Raina et al., 2019).

The CLSA is a large, national, long-term study that follows approximately 50,000 individuals who are between the ages of 45 and 85 when recruited, for at least 20 years. It provides information on the changing biological, medical, psychological, social, lifestyle, and economic aspects of people’s lives. Baseline data reported in this study were collected from data collection sites established in 11 cities across Canada: Victoria, Vancouver, Surrey (British Columbia), Calgary (Alberta), Winnipeg (Manitoba), Hamilton, Ottawa (Ontario), Montreal, Sherbrooke (Quebec), Halifax (Nova-Scotia), and St. John’s (Newfoundland and Labrador). The data set is available to researchers after a Research Ethics Board review.

The present study sought to: (a) examine differences in OSA risk and insomnia between older adults with and without self-reported memory problems; and (b) determine whether OSA risk or insomnia better predicts cognitive slowing, as measured by Choice Reaction Time (CRT).

Based on prior literature, we hypothesized that elevated OSA risk, but not insomnia, would be associated with poorer memory and slower processing speed. Furthermore, we explored whether a distinct clinical profile (e.g., incorporating STOP-BA(N)G score, metabolic status, and demographic factors) could identify individuals at greatest risk for cognitive impairment. Clarifying these associations could enhance early detection of sleep-related cognitive vulnerability and inform preventive strategies for successful aging. Because this study uses baseline CLSA data collected at a single time point, the analyses are inherently cross-sectional and therefore can identify associations but cannot establish temporal or causal relationships.

## 2 Methods

### 2.1 Participants

We selected a subgroup from the CLSA cohort comprising 946 participants (448 females, 498 males; mean age = 64.56, SD = 11.23), recruited from across 10 Canadian provinces (Raina et al., 2009; Raina et al., 2019). Participants who had been told by a doctor that they have memory problems were included in the “Memory problems/yes” group (*n* = 473; 224 females, 249 males; mean age = 64.56, SD = 11.24). An age- and gender-matched group “Memory problems/no” was randomly selected from a pool of 29,443 participants who do not have memory problems using the Case–Control Matching function in SPSS v28 (‘age’ and ‘sex’ as variables to match on / option to maximize execution performance).

### 2.2 Measures

#### 2.2.1 STOP-BANG questionnaire

This is a validated, self-report measure that queries the presence of the following OSA risk factors: snoring, tiredness, observed interruption of breathing during sleep, high blood pressure, body mass index (BMI) more than 35 kg/m^2^, age over 50 years, neck circumference, and gender. Sensitivity using an STOP-BANG score > = 3 is high, while specificity is moderate across OSA severity categories. For the purposes of the present study, this is an acceptable way to identify likelihood of OSA diagnosis that would merit confirmatory testing in a clinical sample. In the CLSA data collection, 7 of the 8 elements of the STOP-BANG questionnaire were identified and collected; neck circumference was not routinely measured but is well known to be highly correlated with body mass index (BMI), which was explicitly measured (Chen et al., 2021). We refer to this measure as “STOP-BA(N)G.” It has been validated as an equivalent estimate for the risk of moderate and severe OSA (Waseem et al., 2022). Based upon the positive answers to the 7 available elements of the STOP-BANG questionnaire. Participants were classified as low risk (score≤2), intermediate risk (score 3–4), and high risk (score≥5), following commonly used clinical cut-offs for the STOP-BANG questionnaire. A recognized limitation of the STOP-BANG instrument is that one point is assigned for male sex, which may underestimate OSA risk in females. To address this potential bias, sex-stratified analyses were conducted and are reported in the Results (Chung et al., 2016).

#### 2.2.2 Insomnia

Insomnia was defined using operational research criteria commonly applied in epidemiological studies when full DSM-5 or ICSD-3 diagnostic interviews are unavailable (Lichstein et al., 2003). All three criteria must be satisfied to qualify as having research-defined insomnia. Specifically, these were assessed via the questions: “Over the last month, how often did it take you more than 30 min to fall asleep?”; “Over the last month, how often did you wake in the middle of the night or too early in the morning and found it difficult to fall asleep again?” (possible responses were: never, less than once per week, once or twice a week, 3–5 times/week, or 6–7 times/week); and “For how long have you had this trouble?.” Groups were then formed according to the presence or absence of insomnia (Yes/No insomnia). COMISA (comorbid insomnia and sleep apnea) was defined as the presence of both research-defined insomnia and intermediate- or high-risk STOP-BA(N)G classification. This operational definition was used because objective sleep-study confirmation of OSA was not available in the CLSA baseline dataset.

#### 2.2.3 Choice reaction time (CRT)

Choice reaction time was a computer-administered test with 60 presentations of two black boxes, with the response stimulus being a smaller white box that appears within one of the black boxes (Woods et al., 2015). Participants were instructed to touch the box that contains the white or colored box as quickly as possible, and then were given an opportunity to practice. Measures generated are latencies and number of correct test trials. Choice reaction times tend to increase and become more variable with age (Hultsch et al., 2002; Williams et al., 2005), increasing for participants over age 50 and then more sharply after age 70 (Der and Deary, 2006; Luchies et al., 2002). An increase in inconsistency in CRT has been found to predict poorer cognitive performance (MacDonald et al., 2003). We chose to examine CRT as it is believed to be associated with cognitive processing speed and working memory function, and as a measure of overall cognitive intactness (Wilhelm and Oberauer, 2006).

#### 2.2.4 Memory problem

Memory problems were assessed using a single self-report item indicating whether a physician had ever told the participant that they had a memory problem (yes/no).

### 2.3 Procedure

The 946 participants selected for this study are from the CLSA comprehensive cohort. All participants completed the CLSA baseline assessment. Data were collected by researchers through in-home, face-to-face interview questionnaires about diet, medication use, chronic disease symptoms, and sleep disorders. Additionally, participants were asked to visit a local data collection site to conduct a neuropsychological assessment, and the collection of data on anthropometrics (i.e., height, weight, waist and hip circumference), and clinical variables (e.g., memory problem, STOP-BA(N)G, sleep, Choice Reaction Time). Research Ethics Board certification was obtained for this project (McGill University IRB# A03-M13-21B / 21/03/048).

### 2.4 Statistical analyses

Using a univariate general linear model, we investigated the association between STOP-BA(N)G results (low, moderate, severe), insomnia (yes/no), memory problems (yes/no), and their interaction. Binary logistic regression analysis was performed to assess the role of descriptive variables and OSA severity with and without insomnia for predicting memory problems. Discriminant analysis was used to verify whether a dominant (i.e., a highly prevalent) sleep profile, could be recognized within Memory Problem yes and Memory Problem No groups separately using hours slept and sleep quality rating duration of insomnia problem as independent variables and Memory Problem yes/no as the grouping variable. Spearman correlations were performed to investigate the association between STOP-BA(N)G scores and sleep parameters (i.e., hours slept, sleep quality rating, duration of insomnia problem) in those with and without memory problems.

All analyses were performed using IBM SPSS Statistics 28.0 with Python Essentials add-on. Continuous variables were checked for normality with the Kolmogorov–Smirnov test. ANOVA tests or the Kruskall-Wallis test assessed differences for continuous variables as well as the appropriateness of the matched group. Post-hoc analysis for multiple comparisons has been performed with Dunn test. Fisher’s exact test or Chi-square tests were performed for differences in categorical variables. Descriptive data are expressed as median (interquartile range, IQR) or frequencies. Clopper-Pearson’s exact test was used for all confidence intervals. Given the multiple statistical tests performed, findings were interpreted conservatively, with emphasis placed on effect sizes and consistency across analyses rather than solely on statistical significance. No formal adjustment for multiple comparisons (e.g., Bonferroni correction) was applied because the study was exploratory in nature and such corrections may increase Type II error. Nevertheless, the primary finding relating STOP-BA(N)G score to memory problems remained significant under conservative adjustment procedures.

## 3 Results

### 3.1 Participant characteristics

Four hundred and seventy-three participants with a memory problem (Memory problem ‘yes’ group) and 473 age- and gender-matched participants without memory problems (Memory problem ‘no’ group) were recruited. There was no difference between groups for age, biological sex, education, and body mass index (BMI). Participants with memory problems had significantly higher odds of having a history of stroke/CVA and diabetes compared with those without memory problems (Stroke: OR = 4.12, 95% CI 2.15–7.89, *p* < 0.001; Diabetes: OR = 1.63, 95% CI 1.20–2.22, *p* = 0.002). Additionally, individuals with higher OSA risk were significantly more likely to report memory problems (OR = 1.52, 95% CI 1.24–1.85, *p* < 0.001) than those with lower OSA risk (Table 1).

**Table 1:** Descriptive variables for participants with and without memory problems.

Descriptive	Memory problems *n* (%)	No memory problems n (%)	OR	95% CI	*p*
Gender					
Female	224 (50.0)	224 (50.0)	1.000	0.775–1.291	1.000
Male	249 (50.0)	249 (50.0)			
Age					
≥65	222 (50.0)	222 (50.0)	1.000	0.775–1.291	1.000
**<**65	251 (50.0)	222 (50.0)			
OSA risk					
Low risk	183 (43)	242 (57)	1.518	1.244–1.853	<0.001
Intermediate risk	231 (54)	196 (46)			
High risk	59 (63)	35 (37)			
BMI					
BMI ≤ 35	407 (87.9)	422 (89.4)	1.161	0.775–1.741	0.469
BMI > 35	56 (12.1)	50 (10.6)			
History of stroke / CVA					
Yes	45 (9.6)	12 (2.5)	0.272	0.141–0.526	<0.001
No	426 (90.4)	461 (97.6)			
History of hypertension					
Yes	216 (46.2)	192 (40.8)	1.053	0.803–1.387	0.699
No	252 (53.8)	279 (59.2)			
History of diabetes					
Yes	128 (27.1)	88 (18.6)	1.469	1.061–2.033	0.020
No	344 (72.9)	385 (81.4)			
Education					
No post-secondary education, or less than a bachelor degree	310 (65.5)	310 (65.5)	1.000	0.765–1.308	1.000
Bachelor’s degree or higher	163 (34.5)	163 (34.5)			

### 3.2 Cognitive functioning in OSA, insomnia, or COMISA

**Figure 1:**
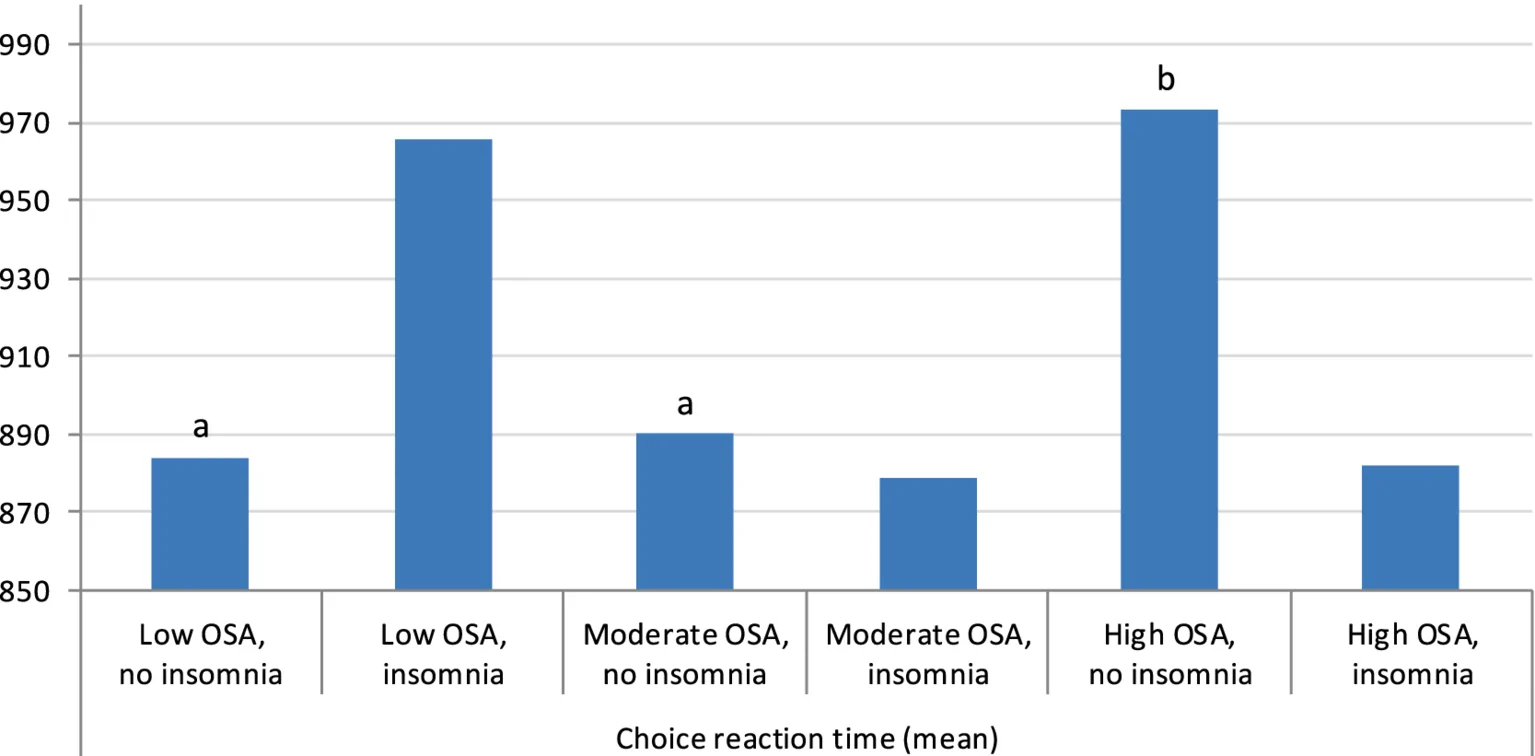
Mean choice reaction time comparing OSA risk and insomnia, *N* = 946.

To assess whether cognitive functioning was worse in participants with COMISA Tables 2–5, Figure 1, we performed a univariate general linear model on STOP-BA(N)G scores using insomnia and memory as variables. Participants with memory problems showed slower CRTs than those without memory problems (Tables 2, 3). With the use of Wilks’s criterion, the model suggested that the combined outcomes were not related to insomnia, *F*(1, 931) = 0.134, *p* = 0.715, but were related to memory problems, *F*(1,931) = 7.103, *p* = 0.008, confirming an Insomnia×Memory interaction being insignificant, *F*(1, 934) = 0.022, *p* = 0.881 (Table 3). Sex-stratified analyses demonstrated that the association between higher STOP-BA(N)G scores and memory problems was present in both men and women. Although effect sizes were somewhat larger among males, the overall pattern of results was consistent across sexes, suggesting that the observed relationship was not solely attributable to the inclusion of male sex within the STOP-BANG scoring system.

**Table 2:** STOP-BA(N)G score means for memory problems yes/no with and without insomnia.

Measure	Memory problems				No memory problems			
	Insomnia		No insomnia		Insomnia		No insomnia	
	M	SD	M	SD	M	SD	M	SD
STOP-BA(N)G score	3.11	1.197	3.07	1.338	2.70	1.287	2.61	1.300

**Table 3:** Fixed-effects ANOVA results using STOP-BA(N)G scores as the criterion.

Predictor	Sum of squares	df	Mean square	*F*	*p*	Partial ŋ^2^
(Intercept)	2131.114	1	2131.114	1235.832	<0.001	0.570
Insomnia	0.231	1	0.231	0.134	0.715	0.000
Memory problems	12.249	1	12.249	7.103	0.008	0.008
Insomnia x memory	0.039	1	0.039	0.022	0.881	0.000
Error	1605.450	931	1.724			

**Table 4:** Choice reaction time (CRT) means for memory problems with and without insomnia.

Measure	Memory problems				No memory problems			
	Insomnia		No insomnia		Insomnia		No insomnia	
	M	SD	M	SD	M	SD	M	SD
Choice reaction time	945.86	265.59	917.31	265.34	852.80	199.58	872.36	307.60

**Table 5:** Fixed-effects ANOVA results using Choice reaction times (CRTs) as the criterion.

Predictor	Sum of squares	df	Mean square	*F*	*p*	Partial ŋ^2^
(Intercept)	205068535.66	1	205068535.66	2537.172	<0.001	0.569
Insomnia	1289.712	1	1289.712	0.016	0.900	0.000
Memory problems	303346.784	1	303346.784	3.753	0.053	0.004
Insomnia x memory	36869.005	1	36869.005	0.456	0.500	0.000
Error	73955453.624	915	80825.632			

To explore the relationship between Insomnia and memory problems, we performed a univariate general linear model (Memory yes/no x Insomnia yes/no) with CRT as the dependent variable. Participants with memory problems showed a trend toward slower CRTs (Tables 4, 5), although this result did not reach conventional statistical significance (*p* = 0.053) (Tables 4, 5). Insomnia was not related to CRT scores.

### 3.3 A risk profile for memory problems

As an attempt to establish a medical profile of patients with memory problems, we performed a discriminant analysis to predict memory problems yes/no). The characteristics of groups that are normally collected by healthcare professionals, with the addition of STOP-BA(N)G scores were used as predictors (age, sex, chronic diseases (hypertension, diabetes), sleep (how many hours of sleep). When applied case-wise to the study population, this equation correctly predicted only 66.6% of cases with memory problems. Nonetheless, correlations between discriminating variables and standardized canonical discriminant functions show that STOP-BA(N)G (*r* = 0.452), and diabetes (*r* = 0.256) accounted for most of the function, Wilks’ Lambda = 0.868, *F* (7, 946) = 130.29, *p* < 0.001.

## 4 Discussion and conclusion

Our findings demonstrate a significant association between OSA risk and cognitive performance among older adults. Participants with memory problems exhibited higher STOP-BA(N)G scores and showed slower CRTs, suggesting reduced processing speed and working-memory efficiency. In contrast, insomnia alone or in combination with OSA (COMISA) was not associated with cognitive outcomes. Together, these findings suggest that OSA risk may be more closely linked to cognitive vulnerability than insomnia symptoms in this population. However, because the study is cross-sectional, no causal inferences can be made.

These results are consistent with prior research reporting associations between OSA and neurocognitive impairment. Importantly, STOP-BA(N)G identifies elevated risk for OSA rather than confirmed disease severity or physiological burden (e.g., apnea-hypopnea index, hypoxia burden). Therefore, mechanisms such as intermittent hypoxia, oxidative stress, endothelial dysfunction, and impaired sleep-dependent restorative processes should be viewed as potential explanatory pathways proposed in the literature rather than direct implications of the present findings. Prior studies have suggested that these mechanisms may contribute to cognitive vulnerability among individuals with OSA.

The higher odds of memory problems among participants at greater OSA risk indicate a meaningful association between sleep-disordered breathing risk and cognitive symptoms. Whether elevated OSA risk contributes to subsequent cognitive decline remains a question for longitudinal investigation. Consistent with the work by Lee et al., these results underscore the importance of examining domain-specific cognitive outcomes—particularly memory functions that may be sensitive to hypoxaemia and sleep fragmentation during critical sleep stages such as (Rapid Eye Movement) REM (Jackson and Rosenzweig, 2025). These associations may reflect disrupted arousal (i.e., vascular coupling or impaired glymphatic clearance), as proposed by Lee et al., emphasizing the need for studies integrating detailed sleep microstructure analysis and advanced neuroimaging markers. Future research that incorporates genetic risk as well as exploration of anti-inflammatory or metabolic interventions may help identify who is most vulnerable to OSA-related cognitive effects and how best to mitigate them.

Epidemiological studies have clearly identified diabetes as a major risk factor for cognitive dysfunction. A novel contribution of this study is the complementary role of OSA risk and the identification of diabetes as a predictor of memory problems. In our discriminant analysis, diabetes and STOP-BA(N)G score together accounted for the majority of the predictive power. This finding supports growing evidence that metabolic dysregulation and sleep-disordered breathing may act synergistically to exacerbate cognitive decline. Both OSA and diabetes are characterized by chronic intermittent hypoxia, systemic inflammation, and impaired glucose metabolism; processes that can accelerate neurodegenerative pathways and compromise cerebrovascular integrity. The coexistence of these conditions may therefore represent a high-risk phenotype for accelerated cognitive aging.

Age-related changes in sleep architecture, such as reduced slow-wave sleep, circadian rhythm alterations, and decreased sleep efficiency, further compound these effects. While such changes are often considered a normal aspect of aging, our findings suggest that when combined with OSA and metabolic comorbidities such as diabetes, they may substantially increase the risk of memory decline and slowed cognitive processing.

Clinically, these findings underscore the importance of integrated screening and management strategies. Older adults presenting with memory complaints should be evaluated not only for OSA but also for diabetes and other cardiometabolic conditions. Conversely, patients with OSA or diabetes should undergo cognitive screening to detect early signs of impairment. Given that effective treatment of OSA (e.g., CPAP or mandibular advancement therapy) and improved glycemic control have both been associated with cognitive benefits, timely intervention could mitigate the progression of cognitive dysfunction.

From a public-health perspective, the implications are significant: OSA remains underdiagnosed in up to 80% of older Canadians, and diabetes prevalence continues to rise with aging. The intersection of these conditions may represent a modifiable target for dementia prevention. Future longitudinal research using CLSA data should explore whether OSA risk and diabetes independently or jointly predict cognitive decline over time, and whether their treatment alters these trajectories.

In summary, this study identifies OSA risk and diabetes as factors associated with cognitive vulnerability in older adults. Screening for both conditions among individuals with memory complaints, and assessing cognition among those with sleep-disordered breathing or metabolic disease, may help identify individuals who could benefit from further evaluation, earlier intervention to reduce neurodegenerative burden, and promote healthier aging across the lifespan. Longitudinal studies are needed to determine whether these factors independently or jointly influence future cognitive decline.

### 4.1 Limitations

The CLSA provides a robust platform for investigating associations between sleep, cognition, and health in a large, nationally representative cohort. However, several limitations should be considered. First, the cross-sectional design precludes conclusions regarding causality or temporal ordering of observed associations. Second, OSA risk was estimated using the modified STOP-BA(N)G questionnaire rather than polysomnography. Although STOP-BA(N)G is a validated screening instrument, it identifies risk rather than confirmed OSA severity. In addition, the instrument assigns one point for male sex, which may underestimate OSA risk in females. Reassuringly, sex-stratified analyses confirmed the association between higher STOP-BA(N)G scores and memory problems in both men and women, although effect sizes were somewhat larger among men (Chung et al., 2016; Chen et al., 2021; Waseem et al., 2022). Third, memory problems were based on a single self-reported physician-diagnosed memory problem item (yes/no), which, while clinically relevant, lacks the precision of multi-item cognitive scales or objective neuropsychological assessments. Future studies should incorporate more comprehensive cognitive measures. Fourth, insomnia was defined using operational research criteria rather than full DSM-5 or ICSD-3 diagnostic interviews, which may affect comparability with clinical diagnoses. Fifth, depressive symptoms and medications with cognitive or sedative effects (e.g., hypnotics, anticholinergic medications) were not comprehensively available within the selected baseline variables and therefore could not be fully controlled. Limited sensitivity analyses using available proxy variables, including self-reported depression diagnosis where available, did not materially alter the primary findings; however, residual confounding cannot be excluded. Sixth, no formal adjustment for multiple comparisons was applied because this study was exploratory, although the principal association between STOP-BA(N)G score and memory problems remained robust under conservative correction approaches. Finally, treatment history, disease duration, and objective indices of OSA severity were unavailable and may influence cognitive outcomes. Despite these limitations, the study provides important evidence that OSA risk is associated with memory complaints and cognitive slowing in a large community-based sample of older Canadians. Future longitudinal analyses incorporating objective sleep assessment, comprehensive neuropsychological testing, and cardiometabolic biomarkers may further clarify these relationships and underlying mechanisms.

## Data Availability

The original contributions presented in the study are included in the article/supplementary material, further inquiries can be directed to the corresponding author.
